# Limitations in predicting PAM50 intrinsic subtype and risk of relapse score with Ki67 in estrogen receptor-positive HER2-negative breast cancer

**DOI:** 10.18632/oncotarget.15748

**Published:** 2017-02-27

**Authors:** Aranzazu Fernand ez-Martinez, Tomás Pascual, Giuseppe Perrone, Serafin Morales, Juan de la Haba, Milagros González-Rivera, Patricia Galván, Francesca Zalfa, Michela Amato, Lucia Gonzalez, Miquel Prats, Federico Rojo, Luis Manso, Laia Paré, Immaculada Alonso, Joan Albanell, Ana Vivancos, Antonio González, Judit Matito, Sonia González, Pedro Fernandez, Barbara Adamo, Montserrat Muñoz, Margarita Viladot, Carme Font, Francisco Aya, Maria Vidal, Rosalía Caballero, Eva Carrasco, Vittorio Altomare, Giuseppe Tonini, Aleix Prat, Miguel Martin

**Affiliations:** ^1^ Medical Oncology Department, Hospital Clínic of Barcelona, Barcelona, Spain; ^2^ Translational Genomics and Targeted Therapeutics in Solid Tumors, August Pi i Sunyer Biomedical Research Institute, Barcelona, Spain; ^3^ Department of Medicine, Università Campus Bio-Medico di Roma, Rome, Italy; ^4^ Medical Oncology Deparment, Arnau de Vilanova de Lleida Universitary Hospital, Lleida, Spain; ^5^ Medical Oncology Department, Reina Sofía University Hospital, Cordoba, Spain; ^6^ Medical Oncology Department, Instituto de Investigación Sanitaria Gregorio Marañón (IISGM), Universidad Complutense, Madrid, Spain; ^7^ Vall d'Hebron Institute of Oncology, Barcelona, Spain; ^8^ Medical Oncology Department, Quirón Hospital, Madrid, Spain; ^9^ Master of Breast Pathology, University of Barcelona, Barcelona, Spain; ^10^ Pathology Department, Fundación Jiménez Díaz Health Research Institute (IIS-FJD), Madrid, Spain; ^11^ Medical Oncology Department, Doce de Octubre Hospital, Madrid, Spain; ^12^ Medical Oncology Department, Hospital del Mar, Barcelona, Spain; ^13^ Medical Oncology Department, MD Anderson Cancer Center, Madrid, Spain; ^14^ Medical Oncology Department, Mutua de Terrassa Hospital, Barcelona, Spain; ^15^ Spanish Breast Cancer Research Group Grupo Español de Investigación en Cáncer de Mama (GEICAM), Madrid, Spain

**Keywords:** PAM50/Prosigna, breast cancer, Ki67, estrogen receptor-positive/HER2-negative

## Abstract

PAM50/Prosigna gene expression-based assay identifies three categorical risk of relapse groups (ROR-low, ROR-intermediate and ROR-high) in post-menopausal patients with estrogen receptor estrogen receptor-positive (ER+)/ HER2-negative (HER2-) early breast cancer. Low risk patients might not need adjuvant chemotherapy since their risk of distant relapse at 10-years is below 10% with endocrine therapy only. In this study, 517 consecutive patients with ER+/HER2- and node-negative disease were evaluated for Ki67 and Prosigna. Most of Luminal A tumors (65.6%) and ROR-low tumors (70.9%) had low Ki67 values (0-10%); however, the percentage of patients with ROR-medium or ROR-high disease within the Ki67 0-10% group was 42.7% (with tumor sizes ≤2 cm) and 33.9% (with tumor sizes > 2 cm). Finally, we found that the optimal Ki67 cutoff for identifying Luminal A or ROR-low tumors was 14%. Ki67 as a surrogate biomarker in identifying Prosigna low-risk outcome patients or Luminal A disease in the clinical setting is unreliable. In the absence of a well-validated prognostic gene expression-based assay, the optimal Ki67 cutoff for identifying low-risk outcome patients or Luminal A disease remains at 14%.

## INTRODUCTION

In the past 10 years, several commercialized multigene prognostic tests have been developed to help guide treatment decisions in patients with early breast cancer [1]. Among them, the PAM50/Prosigna assay (NanoString Technologies, Seattle, WA), identifies the intrinsic molecular subtype (Luminal A, Luminal B, HER2-enriched and Basal-like) and estimates the 10-year risk of relapse (ROR) [2–6] using formalin-fixed paraffin-embedded (FFPE) specimens.

Currently, due to a lack of reimbursement, multigene tests are not readily available for all patients in many countries. Consequently, the use of immunohistochemistry (IHC)-based biomarkers, such as Ki67, has been proposed instead, in order to identify patients with low-risk outcome who may be safely spared chemotherapy [7–9]. However, the 2015 St. Gallen panel proposed that Ki67 scores should be interpreted in light of local laboratory values, and recommended to use the median expression of each lab to define high and low values [9, 10]. In addition, a majority of the panel accepted a threshold value of Ki67 within the range of 20-29%, to distinguish Luminal A from Luminal B disease. These recommendations have led to confusion regarding how to interpret and use Ki67 scoring in the clinical setting.

Here, we aimed to compare the ability of IHC Ki67 to identify those patients at a low risk of recurrence as defined by the clinically and analytically validated commercial version of the PAM50 assay.

## RESULTS

### Cohort characteristics

**Figure 1:**
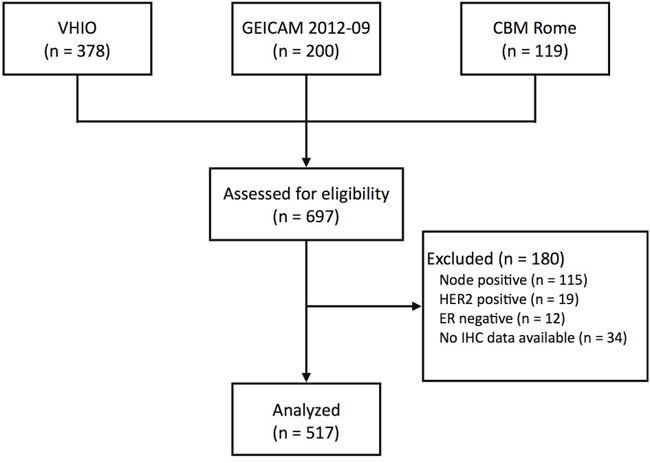
CONSORT diagram VHIO, Vall d’Hebron Institute of Oncology; GEICAM, Spanish Breast Cancer Research Group; CBM Rome, Università Campus Bio-Medico di Roma.

Of the 697 patients, a total of 517 (74.2%) had ER+/HER2-, node-negative disease and Prosigna data available; this cohort was the focus of all further analyses (Figure 1). Prosigna subtype distribution was 56.9% Luminal A, 40.8% Luminal B, 1.2% HER2-enriched, and 1.2% Basal-like (Table 1, Supplementary Table 1). ROR risk group distribution was 38.5% ROR-low, 33.1% ROR-intermediate and 28.4% ROR-high (Supplementary Table 2). Statistically significant differences across the 3 cohorts were observed in ROR-groups but not in subtypes distribution. (Supplementary Tables 1 and 2).

**Table 1:** Distribution of subtypes and ROR within each Ki67 group in 517 patients with HR+/HER2- node-negative disease, ROR-med, ROR-medium

	Ki67 Group			
	0-10%	11-20%	21-30%	>30%
**Intrinsic Subtypes**				
**Luminal A**	193 (81.4%)	63 (51.6%)	29 (29.3%)	9 (15.3%)
**Luminal B**	42 (17.7%)	59 (48.4%)	69 (69.7%)	41 (69.5%)
**HER2-enriched**	2 (0.8%)	0	1 (1.0%)	3 (5.1%)
**Basal-like**	0	0	0	6 (10.2%)
**Total**	237	122	99	59
**ROR and T≤2cm**				
**ROR-Low**	102 (57.3%)	28 (28.9%)	12 (15.2%)	3 (6.5%)
**ROR-Med**	52 (29.2%)	44 (45.4%)	25 (31.6%)	8 (17.4%)
**ROR-High**	24 (13.5%)	25 (28.5%)	42 (53.2%)	35 (76.1%)
**Total**	178	97	79	46
**ROR and T>2cm**				
**ROR-Low**	39 (66.1%)	6 (24.0%)	5 (25.0%)	4 (30.8%)
**ROR-Med**	17 (28.8%)	13 (52.0%)	9 (45.0%)	3 (23.1%)
**ROR-High**	3 (5.1%)	6 (24.0%)	6 (30.0%)	6 (46.2%)
**Total**	59	25	20	13

### Subtype and ROR concordance with Ki67

The concordance rates between Prosigna subtype (i.e. Luminal A vs. others) and IHC subtype (Luminal A-like vs. others) when Ki67 cutoffs of 14% and 20% were used were 70.8% (kappa score = 0.43; moderate agreement) and 69.1% (kappa score = 0.38; weak agreement), respectively. The percentages of Luminal A tumors within Ki67 0-10%, 10-20%, 20-30% and >30% groups were 81.4%, 51.6%, 29.3% and 15.3%, respectively (Table 1 and Supplementary Table 3). The distribution of ROR-low tumors within Ki67 0-10%, 10-20%, 20-30% and >30% groups were 59.5%, 29.7%, 17.2% and 11.9% respectively. (Table 1 and Supplementary Table 4). The percentage of ROR-med/high patients within the Ki67 0-10% group was 42.7% (within tumor size ≤2 cm) and 33.9% (within tumor size > 2 cm) (Table 1 and Supplementary Table 4). Although not all Luminal A tumors are included in the ROR-low group, the ROR-low group is a subset of the Luminal A group and consists of only Luminal A tumors.

### Identification of Luminal A subtype using Ki67

**Figure 2:**
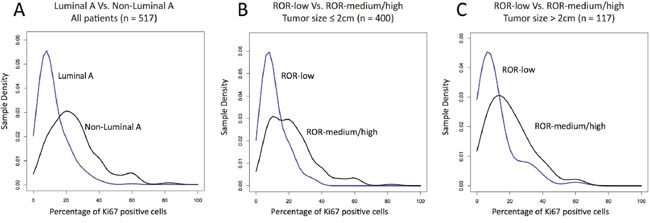
Density of the intrinsic subtypes and ROR-groups based on Ki67-positive cells **(A)** Density plot in Luminal A and non-Luminal A tumors within all patients; **(B)** Density plot of the 3 ROR-groups within tumor sizes ≤2 cm; **(C)** Density plot of the 3 ROR-groups within tumor sizes > 2 cm.

**Figure 3:**
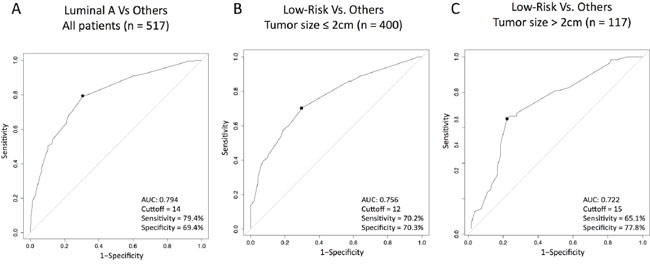
Performance of Ki67 (as a continuous variable) to predict Luminal A or ROR-low disease within HR+/HER2- node-negative disease **(A)** Predicting Luminal A disease (vs. others); **(B)** Predicting ROR-low disease (vs. others) within tumor sizes ≤ 2 cm; **(C)** Predicting ROR-low disease (vs. others) within tumor sizes >2 cm tumors. AUC, area under the curve.

We compared the distribution of Luminal A and non-Luminal A tumors as a function of Ki67 using a density plot (Figure 2A). As expected, Luminal A tumors were more represented within low Ki67 scores and non-Luminal A tumors were more represented within high Ki67 scores, although considerable overlap was observed. To try to identify an optimal Ki67 cutoff to discriminate Luminal A versus non-Luminal A, we estimated the performance of Ki67 (as a continuous variable). The result revealed an area under the receiver operating characteristic (auROC) curve of 0.79 and an optimal cutoff of 14% (Figure 3A). It is noteworthy to highlight that this is practically the same Ki67 cutoff reported by the original work by Cheang and colleagues [11], where PAM50 quantitative real time polymerase chain reaction (qRT-PCR) based subtyping was compared to Ki67 data for the first time.

### Identification of ROR-low using Ki67

Similar to subtype identification, we compared the distribution of ROR-low, ROR-intermediate and ROR-high as a function of Ki67 using 2 density plots, one within tumor sizes ≤2 cm (Figure 2B) and the other one within tumor sizes above 2 cm (Figure 2C). As expected, ROR-low tumors were more represented within low Ki67 scores and ROR-intermediate/high tumors were more represented within high Ki67 scores, although considerable overlap was observed. To try to identify an optimal Ki67 cutoff to discriminate ROR-low versus ROR-intermediate/high, we estimated the performance of Ki67 (as a continuous variable) to identify both groups. The results revealed auROC curves within tumor sizes of ≤2 cm and >2 cm of 0.76 and 0.72, respectively (Figure 3B-3C). The optimal Ki67 cutoffs for identifying ROR-low samples within tumor sizes of ≤2 cm and >2 cm were 12% and 15%, respectively.

### Identification of Luminal A or ROR-low disease using ER and PR levels

**Figure 4:**
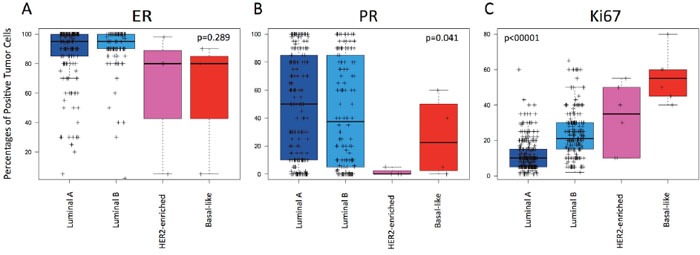
Levels of estrogen receptor (ER), progesterone receptor (PR) and Ki67-positive cells across the intrinsic subtypes within HR+/HER2-negative node-negative disease **(A)** ER; **(B)** PR; **(C)** Ki67. P-values were calculated by comparing mean values across all groups.

Finally, we evaluated if the quantitative expression of ER and PR by IHC could help identify either Luminal A. None of the two IHC-based biomarkers was found useful (Figure 4). However, non-Luminal subtypes (i.e. HER2-enriched and Basal-like combined) showed statistically significant lower ER (62.6% vs 88.6%, p-value=0.003), lower PR (13.75% vs 46.5%, p-value=0.016) and higher Ki67 (43.1% vs 16.18%, p-value<0.001), respectively, compared to both luminal subtypes combined.

## DISCUSSION

To our knowledge, this is the first report that compares ROR and subtype prediction using Prosigna and Ki67 in the same sample set. Our results highlight the important discrepancy between both biomarkers, and challenge the notion that gene expression-based assays are not needed in patients with HR+/HER2- disease with either low (i.e. <10%) or high (i.e. >20%) Ki67 scores.

The prognostic ability of Prosigna assay has been tested in samples from two phase III clinical trials, Arimidex, Tamoxifen, Alone or in Combination trial (ATAC) and Austrian Breast & Colorectal Cancer Study Group 08 (ABCSG08) [3, 12], involving a total of 2,485 post-menopausal patients treated with adjuvant endocrine therapy alone for 5 years. The results showed that Prosigna assay can identify a group of patients who do not need adjuvant chemotherapy due to their low risk (i.e. <10%) of distant recurrence at 10 years with endocrine therapy administered only [3, 4]. Moreover, Prosigna ROR score and intrinsic subtypes are predictors of late recurrence [5, 13] and response to multi-agent chemotherapy in the neoadjuvant setting [14]. In the recently reported American Society of Clinical Oncology (ASCO) Clinical Practice Guidelines, Prosigna was identified as an assay with the highest level of evidence to guide decisions on adjuvant systemic therapy in patients with ER+/HER2- and node-negative tumors [15].

In 2009, Cheang et al. [11] compared Ki67 and gene expression, using the qRT-PCR-based PAM50 version, and identified 13.25% as the optimal Ki67 cutoff to identify Luminal A versus Luminal B disease. The authors noted that despite this result, the sensitivity and specificity was around 75%, meaning that 1 out of 4 patients evaluated would not be classified correctly. With similar sensitivity and specificity (79.4% and 69.4% respectively), our study confirms that ∼14% is an optimal cutoff for identifying low risk outcome patients who can be spared adjuvant chemotherapy when gene expression-based assays are not available.

In our view, our findings are important as much as it places the Ki67 cutoff at 14%; in 2013 St. Gallen International Expert Consensus proposed a Ki67 cutoff of 20% together with tumor size and nodal status to help identify low risk patients [8], and the 2015 St. Gallen panel recommended to use the median expression of Ki67 of each lab to define high and low Ki67 values [9, 10]. Although recommendations from the international Ki67 in breast cancer working group have led to improvements in reproducing of Ki67 [16], several studies have reported a high inter-laboratory variability in Ki67 scoring [17, 18].

Our study has several limitations. First, we do not have survival outcome data. Thus, we cannot compare the true prognostic value of the discrepant cases between the two assays. However, the level of analytical and clinical validation of the Prosigna assay to identify low-risk outcome patients, or Luminal A disease, is higher than the levels of validation of Ki67. According to Simon et al. criteria [19], Ki67 has not reached level 1 evidence mainly due to the suboptimal inter-laboratory reproducibility and the lack of a clinically useful cutoff [20]. Second, the IHC assessment of Ki67 was done using three different assays across the three cohorts of the study. However, the results regarding performance and the optimal Ki67 cutoff were not affected when adjusted for each type of cohort (Supplementary Figures 1, 2 and 3 and Supplementary Tables 5, 6 and 7). Third, the number of samples in the group of patients with tumors > 2 cm was low.

To conclude, although Ki67 has repeatedly shown to be prognostic [21, 22] and predictive of chemotherapy response [23, 24], the clinical value of Ki67 in identifying low risk outcome patients or Luminal A disease who might be safely spared chemotherapy remains uncertain. In absence of a well-validated prognostic gene expression-based assay, the optimal Ki67 cutoff in identifying low risk outcome patients (together with tumor size and nodal status) or Luminal A disease remains at 14%. However, it is worth highlighting that ∼50% of patients with Luminal A-like disease (e.g. ER+/PR>20%/HER2- and Ki67<14%), node-negative and a tumor size above 2 cm, will not be classified as ROR-low.

## MATERIALS AND METHODS

### Cohorts of patients

Prosigna and IHC data were evaluated from 3 independent cohorts (Spanish Breast Cancer Research Group GEICAM/2012-09 prospective study [25], Vall d’Hebron Institute of Oncology [VHIO] Translational Genomics Lab and Campus Bio-Medico University of Rome [CBM-Rome] Molecular Diagnostic Lab) with a total of 697 consecutive postmenopausal women with early breast cancer (Figure 1). The GEICAM/2012-09 was a prospective study of the Spanish Breast Cancer Research Group to characterize the impact of Prosigna assay in adjuvant treatment decision of 200 postmenopausal patients with ER+/HER2- breast cancer without nodal involvement [25]. VHIO and CBM-Rome tested 378 and 119 independent tumor samples (as of November 31^st^, 2016) coming from patients treated in clinical practice in Spain and Italy and whose medical oncologist decided to order a Prosigna® assay. Similar to GEICAM 2012-09 study, we selected patients with ER-positive/HER2-negative early breast cancer without nodal involvement. All procedures were performed in accordance with the ethical standards of the institutional and/or national research committee and with the 1964 Helsinki declaration ethical standards. Informed consent was obtained from all individual participants included in the study.

### Immunohistochemistry

IHC data was obtained from either central review (GEICAM/2012-09 and CBM-Rome) or from medical reports (VHIO) sent to the pathology laboratory. Ki67 was assessed by IHC using CONFIRM anti-Ki67 (30-9) Rabbit Monoclonal Primary Antibody (Ventana Medical System) in the GEICAM/2012-09 cohort. Anti-Ki67 MIB1 clone antibody (Dako, Glostrup, Denmark) was used in the CBM-Rome cohort. No data on Ki67 assessment is available for VHIO samples since Ki67 determinations were done in multiple local labs. In all samples from GEICAM/2012-09 and Campus Bio-Medico, Ki67 interpretation criteria were done according to the latest international recommendations [16].

We defined Luminal A-like or Luminal B-like tumors according to the IHC surrogate definitions of breast cancer subtypes proposed in the 13^th^ St Gallen International Breast Cancer Conference [9]: Luminal A-like tumors were defined as HER2-negative, ER-positive with a low Ki67 assessment (<14%) and Luminal B-like tumors were defined as HER2-, ER-positive with a high Ki67 determination (≥14%). Tumors with a low-Ki67 determination (<14%) were considered as Luminal B-like tumors if PR was <20% (when PR was available) [16]. A cutoff of 20% of Ki67 was also explored.

### Prosigna assay

FFPE tumors were analyzed using the commercialized and standardized PAM50/Prosigna assay (NanoString Technologies, Seattle, WA) [1–6]. We have followed the specifications of the package insert 2015-07 LBL-C0223-05.

### Statistical analysis

All statistical analyses were performed using R version 3.2.2 (www.r-project.org). We used the Cohen's kappa coefficient to analyze the agreement between IHC-subtypes and Prosigna-subtypes. Quantitative data from visual assessment of Ki67 IHC determination (as a continuous variable) was compared against Luminal A and ROR-low groups as defined by Prosigna. The optimal cutoff value for Ki67 was selected by using the auROC method and maximizing the Youden index (the sum of sensitivity and specificity minus one).

## SUPPLEMENTARY MATERIALS FIGURES AND TABLES



## Abbreviations

ROR: risk or recurrence
ER: estrogen receptor
HER2: human epidermal growth factor receptor 2
FFPE: formalin-fixed paraffin-embedded
IHC: immunohistochemistry
auROC: area under the receiver operating characteristic curve
qRT-PCR: quantitative real time polymerase chain reaction
PR: progesterone receptor
ATAC: Arimidex, Tamoxifen, Alone or in Combination Clinical Trial
ABCSG08: Austrian Breast & Colorectal Cancer Study Group 08 ROR
ASCO: American Society of Clinical Oncology
GEICAM: Spanish Breast Cancer Research Group
VHIO: Vall d’Hebron Institute of Oncology
