# Supplementary material for: Limitations in predicting PAM50 intrinsic subtype and risk of relapse score with Ki67 in estrogen receptor-positive HER2-negative breast cancer
Source: Oncotarget. 2017 Feb 27;8(13):21930–7. doi: 10.18632/oncotarget.15748 (PMC5400635; doi:10.18632/oncotarget.15748)
Supplement: Supplementary file 1 [file oncotarget-08-21930-s001.pdf]

Limitations in predicting PAM50 intrinsic subtype and risk of relapse score with Ki67 in estrogen receptor-positive HER2-negative breast cancer

SUPPLEMENTARY MATERIALS

SUPPLEMENTARY FIGURES AND TABLES

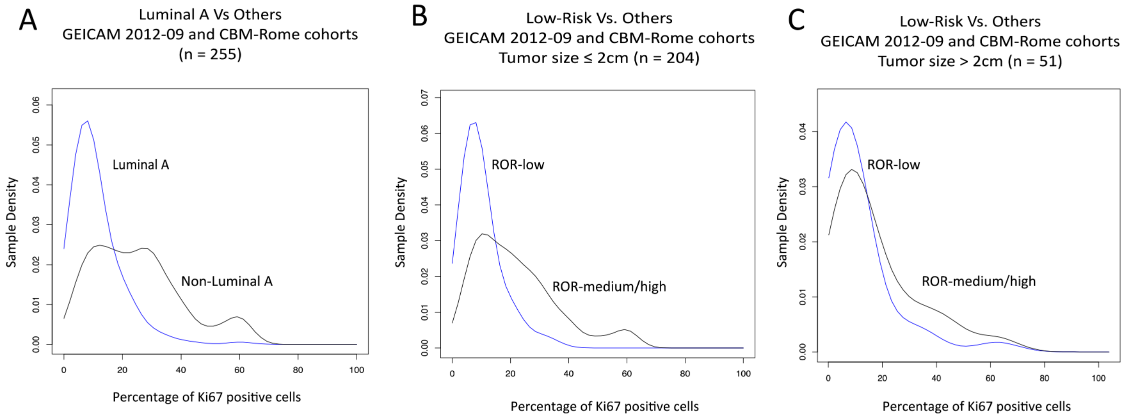

Supplementary Figure 1: Density of the intrinsic subtypes and ROR-groups based on Ki67 positive cells in GEICAM 2012-09 and CBM-Rome cohorts. **(A)** Density plot in Luminal A and non-Luminal A tumors within all patients; **(B)** Density plot of the ROR-groups within tumor sizes ≤2 cm; **(C)** Density plot of the ROR-groups within tumor sizes > 2 cm.

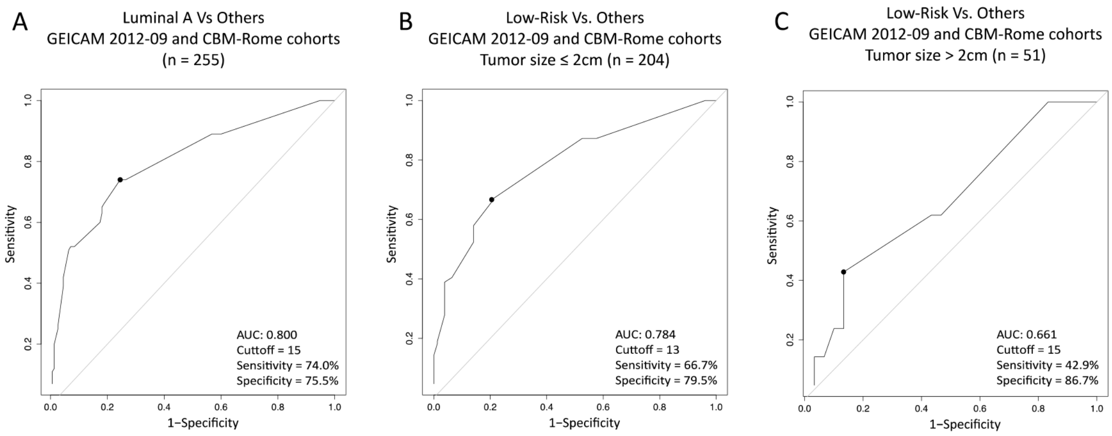

Supplementary Figure 2: Performance of Ki67 (as a continuous variable) to predict Luminal A or ROR-low disease within HR+/HER2-negative node-negative disease (GEICAM 2012-09 and CBM-Rome cohorts). **(A)** Predicting Luminal A disease (vs. others); **(B)** Predicting ROR-low disease (vs. others) within tumor sizes ≤ 2 cm; **(C)** Predicting ROR-low disease (vs. others) within tumor sizes >2 cm tumors. AUC, area under the curve.

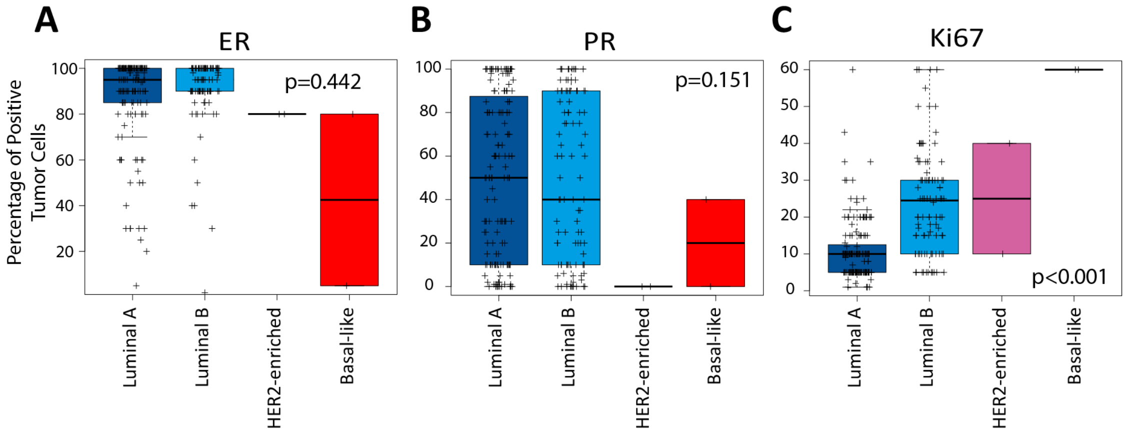

Supplementary Figure 3: Levels of estrogen receptor (ER), progesterone receptor (PR) and Ki67-positive cells across the intrinsic subtypes within HR+/HER2-negative node-negative disease (GEICAM 2012-09 and CBM-Rome cohorts). **(A)** ER; **(B)** PR; **(C)** Ki67. P-values were calculated by comparing mean values across all groups.

Supplementary Table 1: Distribution of the intrinsic subtypes within HR+/HER2-negative and node-negative disease across the 3 cohorts

	VHIO	GEICAM 2012-09	CBM Rome	All patients
**Lum A**	139 (53.1%)	108 (67.1%)	47 (50.0%)	294 (56.9%)
**Lum B**	115 (43.9%)	50 (31.1%)	46 (48.9%)	211 (40.8%)
**HER2-enriched**	4 (1.5%)	2 (1.2%)	0	6 (1.2%)
**Basal-like**	4 (1.5%)	1 (0.6%)	1 (1.1%)	6 (1.2%)
**N**	262	161	94	517

Chi-square 12.404 p = 0.054.

Supplementary Table 2: Distribution of the ROR groups within HR+/HER2-negative and node-negative disease across the 3 cohorts

	VHIO	GEICAM 2012-09	CBM Rome	All patients
**Low risk**	91 (34.7%)	83 (51.6%)	25 (26.6%)	199 (38.5%)
**Intermediate risk**	85 (32.4%)	51 (31.7%)	35 (37.2%)	171 (33.1%)
**High risk**	86 (32.8%)	27 (16.8%)	34 (36.2%)	147 (28.4%)
**N**	262	161	94	517

Chi-square 23.630 p = 0.000095.

Supplementary Table 3: Distribution of subtypes across each Ki67 group in 517 patients with HR+/HER2-negative node-negative disease

	Intrinsic subtypes			
	**Luminal A**	**Luminal B**	**HER2-enriched**	**Basal-like**
**Ki67 group**				
0-10%	193 (65.6%)	42 (19.9%)	2 (33.3%)	0
11-20%	63 (21.4%)	59 (28.0%)	0	0
21-30%	29 (9.9%)	69 (32.7%)	1 (16.7%)	0
>30%	9 (3.1)	41 (19.4%)	3 (50%)	6 (100%)
**Total**	294	211	6	6

Supplementary Table 4: Distribution of ROR across each Ki67 group in 517 patients with HR+/HER2-negative node-negative disease

	ROR and T≤2cm		
	**ROR-Low**	**ROR-Med**	**ROR-High**
**Ki67 group**			
0-10%	102 (70.3%)	52 (40.3%)	24 (19.0%)
11-20%	28 (19.3%)	44 (34.1%)	25 (19.8%)
21-30%	12 (8.3%)	25 (19.4%)	42 (33.3%)
>30%	3 (2.1%)	8 (6.2%)	35 (27.8%)
**Total**	145	129	126
	**ROR and T>2cm**		
	**ROR-Low**	**ROR-Med**	**ROR-High**
**Ki67 group**			
0-10%	39 (72.2%)	17 (40.5%)	3 (14.2%)
11-20%	6 (11.1%)	13 (31.0%)	6 (28.6%)
21-30%	4 (9.3%)	9 (21.4%)	6 (28.6%)
>30%	4 (7.4%)	3 (7.1%)	6 (28.6%)
**Total**	54	42	21

Supplementary Table 5: Distribution of subtypes and ROR within each Ki67 group in 255 patients with HR+/HER2-negative node-negative disease (GEICAM 2012-09 and CBM-Rome cohorts)

	Ki67 Group			
	**0-10%**	**11-20%**	**21-30%**	**>30%**
**Intrinsic Subtypes**				
Luminal A	114 (81.4%)	28 (56%)	9 (25.7%)	4 (13.3%)
Luminal B	25 (17.9%)	22 (44%)	26 (74.3%)	23 (76.7%)
HER2-enriched	1 (0.7%)	0	0	1 (3.3%)
Basal like	0	0	0	2 (6.7%)
**Total**	140	50	35	30
**ROR and T≤2cm**				
**ROR-Low**	61 (59.8%)	12 (26.1%)	4 (12.9%)	1 (4%)
**ROR-Med**	30 (29.4%)	22 (47.8%)	12 (38.7%)	4 (16%)
**ROR High**	11 (10.8%)	12 (26.1%)	15 (48.4%)	20 (80%)
**Total**	102	46	31	25
**ROR and T>2cm**				
**ROR-Low**	26 (68.4%)	0	2 (50%)	2 (40%)
**ROR-Med**	12 (31, 6%)	3 (75%)	2 (50%)	1 (20%)
**ROR-High**	0	1 (25.0%)	0	2 (40%)
**Total**	38	4	4	5

Supplementary Table 6: Distribution of subtypes across each Ki67 group in 255 patients with HR+/HER2-negative node-negative disease (GEICAM 2012-09 and CBM-Rome cohorts)

	Intrinsic subtypes			
	**Luminal A**	**Luminal B**	**HER2-enriched**	**Basal-like**
**Ki67 group**				
0-10%	114 (73.5%)	25 (26.0%)	1 (50.0%)	0
11-20%	28 (18.1%)	22 (22.9%)	0	0
21-30%	9 (5.8%)	26 (27.1%)	0	0
>30%	4 (2.6%)	23 (24.0%)	1 (50.0%)	2 (100%)
**Total**	155	96	2	2

Supplementary Table 7: Distribution of ROR across each Ki67 group in 255 patients with HR+/HER2-negative node-negative disease (GEICAM 2012-09 and CBM-Rome cohorts)

	ROR and T≤2cm		
	**ROR-Low**	**ROR-Med**	**ROR-High**
**Ki67 group**			
0-10%	61 (78.2%)	30 (44.1%)	11 (19.0%)
11-20%	12 (15.4%)	22 (32.4%)	12 (20.7%)
21-30%	4 (5.1%)	12 (17.6%)	15 (25.9%)
>30%	1 (1.3%)	4 (5.9%)	20 (34.5%)
**Total**	78	68	58
	**ROR and T>2cm**		
	**ROR-Low**	**ROR-Med**	**ROR-High**
**Ki67 group**			
0-10%	26 (86.7%)	12 (66.7%)	0
11-20%	0	3 (16.7%)	1 (33.3%)
21-30%	2 (6.7%)	2 (11.1%)	0
>30%	2 (6.7%)	1 (5.6%)	2 (66.7%)
**Total**	30	18	3
